# A 3D-Printed Home-Based Arthroscopic Simulator Improves Basic Surgical Skills: A Prospective Comparative Multicentre Study

**DOI:** 10.3390/jfmk11010126

**Published:** 2026-03-21

**Authors:** Marco Montemagno, Luigi Zaffarana, Flora Maria Chiara Panvini, Ludovico Lucenti, Alessandra Di Nora, Egidio Avarotti, Angelo Di Giunta, Gianluca Testa, Vito Pavone

**Affiliations:** 1 Department of General and Special Surgery, Section of Orthopaedics, University Hospital Policlinico G. Rodolico-San Marco, University of Catania, 95123 Catania, Italy; docmontemagno@gmail.com (M.M.); flo.panvini@gmail.com (F.M.C.P.); gianpavel@hotmail.com (G.T.); 2 Orthopaedics and Traumatology Unit, ARNAS Garibaldi–Centro, 95124 Catania, Italy; 3 STMicroelectronics, 95121 Catania, Italy; luigi.zaffarana@gmail.com; 4 Department of Precision Medicine in Medical, Surgical and Critical Care (Me.Pre.C.C.), University of Palermo, 90127 Palermo, Italy; ludovico.lucenti@gmail.com; 5 Orthopaedic Division of Policlinico “G.B. Morgagni”, 95125 Catania, Italy; adigiunta@yahoo.com

**Keywords:** arthroscopy, simulator, surgical education, orthopaedic training, low-cost innovation

## Abstract

**Objectives**: Arthroscopic surgery requires complex visuospatial coordination and psychomotor skills, which are traditionally acquired through mentorship and cadaveric training. High-fidelity simulators are effective but often costly and inaccessible. This study evaluates the technical effectiveness of a novel home-based 3D-printed arthroscopic simulator (“Arthrozero”) for improving basic arthroscopic skills among orthopedic residents. **Methods**: Thirty-three orthopedic residents (25–36 years) from two Italian university centers were randomized into three groups: ZERO (Arthrozero training), ARTHRO (real arthroscope training), and CONTROL (theoretical session). Training was performed on a FAST-like workstation through four progressively complex tasks. Performance metrics included task completion time, number of looks down, and skill progression during a final Shoulder Challenge (SHO-CHA) assessment. A web-based Likert questionnaire evaluated participant satisfaction and perceived educational value. **Results**: Both ZERO and ARTHRO groups demonstrated significant improvement across training sessions (*p* < 0.05) for all tasks, while the CONTROL group showed minimal gains. In the SHO-CHA assessment, mean completion times were 394.1 ± 140.7 s (ZERO), 456.1 ± 123.2 s (ARTHRO), and 745.5 ± 190.7 s (CONTROL) (*p* < 0.01). No significant difference was observed between ZERO and ARTHRO groups (*p* = 0.276). **Conclusions**: The home-based Arthrozero simulator demonstrated improvements in basic arthroscopic skill performance, suggesting that it may represent an accessible training tool to support early arthroscopic skill acquisition alongside traditional training methods.

## 1. Introduction

Arthroscopic surgery represents one of the most significant advancements in modern orthopedic practice, providing minimally invasive diagnostic and therapeutic options that improve patient recovery and reduce surgical morbidity [1,2]. Over the past decades, technological developments in optics, digital imaging, and instrumentation have expanded the indications and applications of arthroscopy across multiple joints, including the knee, shoulder, hip, wrist, and ankle [3,4,5,6,7,8,9,10,11].

Despite these technological advances, the acquisition of arthroscopic skills remains challenging for trainees due to the unique psychomotor and visuospatial demands associated with video-assisted procedures.

Unlike open surgery, arthroscopy requires surgeons to operate using a two-dimensional monitor while manipulating instruments within a three-dimensional environment. The loss of binocular vision, reduced tactile feedback, the ability to interpret indirect visual feedback while performing precise movements, the fulcrum effect of portals, and the need for triangulation create a complex learning process characterized by prolonged training periods and steep learning curves [12,13,14]. Arthroscopic competence typically requires repeated exposure, with studies suggesting that between 20 and 40 supervised procedures may be necessary to achieve basic proficiency, while more complex interventions may require 50–100 cases or more [15,16]. These challenges highlight the importance of structured training strategies that allow safe and repetitive practice before exposure to real surgical environments.

Modern surgical education has progressively shifted toward competency-based training models, emphasizing deliberate practice, objective assessment, and simulation-based learning [17,18]. The implementation of structured technical skill evaluation tools, such as OSATS and other objective assessment systems, has reinforced the importance of measurable skill acquisition during residency training [17,19,20,21]. As a result, alternative training modalities that provide standardized and reproducible practice opportunities have become increasingly relevant.

Simulation-based education has emerged as a fundamental component of surgical training, allowing residents to develop psychomotor skills in a safe and controlled environment without risk to patients [22,23,24]. Evidence suggests that simulation can facilitate skill acquisition and may contribute to measurable improvements in technical performance before exposure to the operating room environment [23,25]. Both virtual reality and physical simulation models have been introduced to replicate arthroscopic tasks and improve technical competence among trainees [26,27,28]. However, the effectiveness of high-fidelity virtual simulators remains debated, with some studies reporting limited construct validity or inconsistent correlation between simulator performance and surgical experience [29,30].

Besides concerns about educational effectiveness, the adoption of advanced simulation technologies is frequently limited by high costs, limited availability, and the need for dedicated training facilities. Consequently, there has been growing interest in low-cost and home-based simulation solutions that allow repeated practice and self-directed learning [31,32,33,34]. Box trainers and non-anatomic physical models have demonstrated potential for developing triangulation and basic instrument handling skills, while anatomic models provide more realistic procedural environments [35,36,37].

However, many lack realistic arthroscopic optical characteristics, such as a 30° viewing angle, ergonomic handling, and instrument manipulation similar to a real surgical arthroscope [33,36,38,39]. This limitation may reduce their educational effectiveness and limit the transferability of acquired skills to real clinical scenarios. Therefore, the development of accessible, technically realistic, and portable arthroscopic simulation devices remains a relevant educational need.

The aim of this prospective comparative study was to evaluate the educational effectiveness of a low-cost, home-based arthroscopic simulator (“Arthrozero”) designed to improve basic psychomotor skills, including hand–eye coordination, triangulation, and probing ability in trainee surgeons. We hypothesized that participants trained using the home-based Arthrozero simulator would demonstrate improvements in basic arthroscopic task performance compared with untrained controls, and that their performance would approach that observed in participants exposed to traditional arthroscopic training.

## 2. Materials and Methods

### 2.1. Study Design and Participants

This experimental study was designed as a prospective, randomized, comparative, multicenter trial. Orthopedic residents aged between 25 and 36 years were recruited from two University Residency Programs in Southern Italy. Inclusion criteria consisted of no prior active experience as a first operator in arthroscopic surgery. Exclusion criteria included any active participation as a primary operator in arthroscopic procedures.

All participants voluntarily agreed to take part in the study and were naïve to formal arthroscopic simulation training at the time of enrollment.

### 2.2. Data Collection and Questionnaires

For each participant, demographic and background variables were collected, including sex, age (years), postgraduate year of residency (PGY), number of arthroscopic procedures observed as an assistant or observer, type of manual activities performed (e.g., sports involving hand use, traditional or digital arts, video gaming, manual hobbies), and level of interest in arthroscopic surgery, assessed on a progressive Likert scale from 1 to 5.

After completion of the arthroscopic testing sessions, all participants completed a digital, web-based satisfaction questionnaire using a Likert scale. The questionnaire investigated the following aspects:

- perceived usefulness of the arthroscopic training modality;
- overall satisfaction with the training experience;
- perceived differences between the “Arthrozero” simulator and a real surgical arthroscope;
- perceived ability of the simulator to replace or supplement traditional arthroscopic training.

### 2.3. Arthrozero Simulator Prototyping

**Figure 1:**
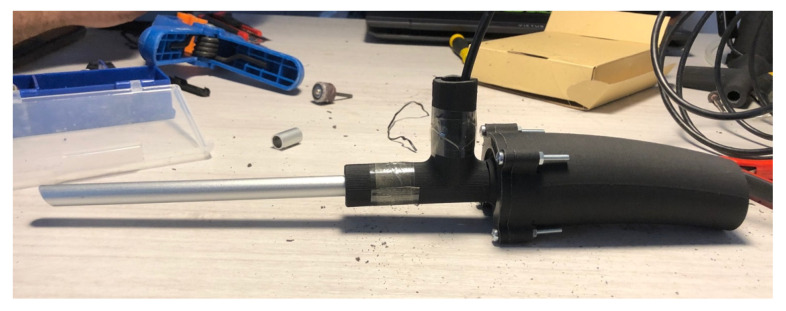
Arthrozero (original design prototype) profile view.

“Arthrozero” is a custom-designed, home-based arthroscopic simulator developed for this experimental study. The device was manufactured using lightweight 3D-printed PLA (polylactic acid, eSUN Ltd., Shenzhen, China) material and was designed to replicate the ergonomics, grip, and dimensions of a standard surgical arthroscope (Figure 1).

The simulator incorporates an internally embedded electronic system equipped with a 30° digital endoscopic camera, allowing 360° orientation inside the simulator chamber, mounted within an 8 mm aluminum cannula with an integrated light source, closely resembling a real arthroscopic lens rod. Video output is transmitted via a USB (Universal Serial Bus) connection and displayed on a laptop monitor. An open-source software platform allows real-time visualization while preserving telescoping and periscoping functions analogous to those of an analog arthroscope. To substantiate the “low-cost” and scalability claims, an approximate cost breakdown of the Arthrozero simulator was calculated. The 3D-printed component required approximately 56 g of PLA filament; the material cost per unit was approximately 1.5 EUR. Printing time was approximately 2.5 h, with an estimated electricity consumption cost of approximately 2–3 EUR. The aluminum cannula used in the device costs approximately 5 EUR, and the microcamera with integrated light source costs approximately 15 EUR. The visualization software was open-source and therefore incurred (VLC media player, v. 3.0.2) no additional cost. Overall, the estimated total production cost per unit was approximately 25 EUR. These features support the economic accessibility and scalability of the proposed simulator for home-based training and institutional implementation.

These technical characteristics, combined with the original ergonomic design, differentiate Arthrozero from previously described low-cost arthroscopic simulators and allow a closer approximation to the optical and handling features of a real arthroscope.

### 2.4. Training Environment and Arthroscopic Tasks

Arthroscopic training was performed using an adaptation of the validated FAST workstation (Fundamentals of Arthroscopy Surgery Training module; SAWBONES^®^, Pacific Research Laboratories, Vashon, WA, USA), consisting of a box-type arthroscopic trainer with an opaque dome [40]. This workstation is designed to train fundamental arthroscopic skills, including telescoping, periscoping, triangulation, probing, and object manipulation.

Specifically, the FAST-like modules used in this study addressed the following skills:

- Telescoping, assessing camera guidance and horizon adjustment;
- Periscoping, training 360° camera tracking, correct use of a 30° arthroscope, object centering, and control of the viewing direction;
- Triangulation, focusing on coordinated movements between the camera and probe;
- Probing, training fine motor control on mobile or stationary targets;
- Manipulation, involving object grasping, pulling, and controlled movement.

**Figure 2:**
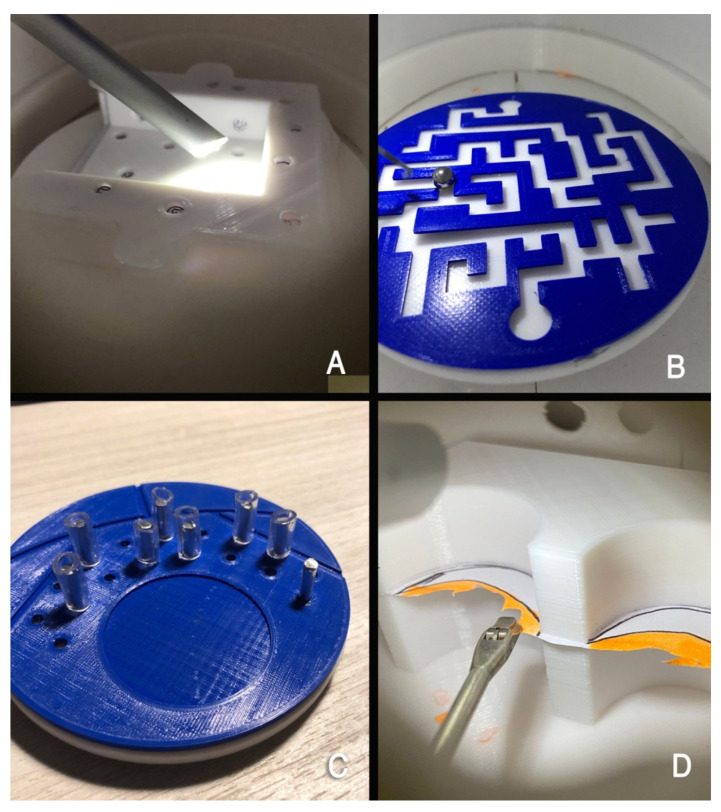
FAST-like workstation training modules used in the study for arthroscopic skill development. (**A**) Number-finding module; (**B**) Labyrinth navigation module; (**C**) Cylinder insertion module; (**D**) Meniscectomy module.

Four tasks of increasing difficulty were selected based on basic and advanced motor skills proposed by combined guidelines from the Arthroscopy Association of North America (AANA), the American Academy of Orthopaedic Surgeons (AAOS), and the American Board of Orthopaedic Surgery (ABOS) [41]. The workstation modules included (Figure 2):

1. Progressive number-finding module (telescoping and periscoping skills);
2. Labyrinth navigation module (telescoping and probing skills);
3. Eight-cylinder insertion module (manipulation skills);
4. Partial medial and lateral meniscectomy module (advanced surgical manipulation skills).

### 2.5. Training Protocol and Assessment Methods

Participants were randomized and allocated into three groups using a computer-generated randomisation sequence with a 1:1:1 allocation ratio. Allocation was performed by an investigator not involved in outcome assessment. The first group (ZERO) underwent two arthroscopic training sessions using the Arthrozero simulator, each lasting 30 min, performing the four FAST tasks described above. The second group (ARTHRO) underwent two 30 min training sessions using a standard surgical arthroscope on the same FAST-like workstation, performing identical tasks. The third group (CONTROL) received a single theoretical session consisting of a general presentation on the basic principles of arthroscopy. The CONTROL group did not participate in practical simulator training. This design was intended to provide a baseline comparison with participants undergoing structured hands-on practice using the Arthrozero or conventional arthroscopic training setups. Due to the nature of the intervention, participant blinding was not feasible. However, the evaluator responsible for measuring task completion time was blinded to group allocation. Two 30 min training sessions were selected to standardize exposure across participants and to simulate a realistic and reproducible training format compatible with residency schedules. This duration was considered sufficient to evaluate early skill acquisition and short-term learning progression, while avoiding fatigue-related performance bias. Participants were explicitly instructed not to perform additional arthroscopic simulator practice between sessions, and no protocol deviations were reported. No unsupervised or extra training was reported during the study period.

**Figure 3:**
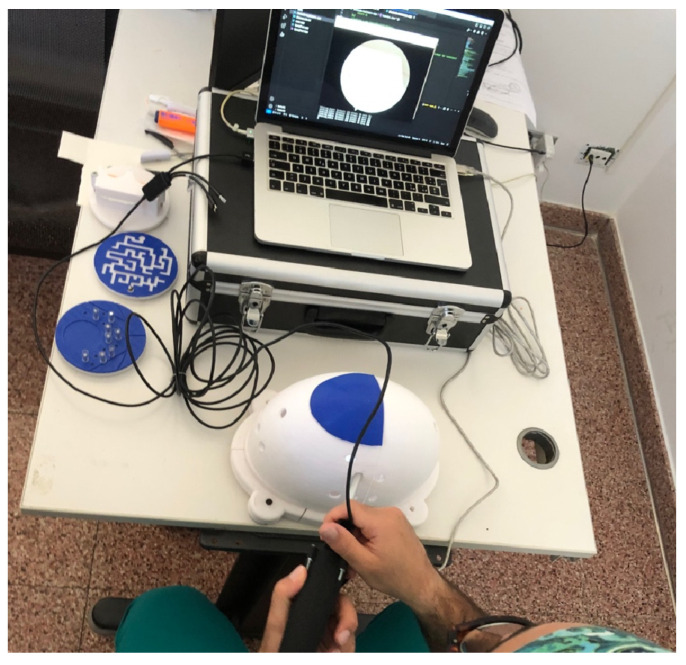
Arthrozero training session (ZERO group) performed on the FAST-like training module used in the study, illustrating the simulator setup and participant interaction during the task execution.

Following a standardized demonstration of the four FAST tasks, participants in the ZERO and ARTHRO groups performed the exercises during separate sessions using standard arthroscopic portals (anteromedial, anterolateral, and anterior portals when required) (Figure 3). Equipment setup, platform positioning, and instruments were standardized across all sessions.

For the ZERO group, training was performed using the Arthrozero simulator, a 5 mm probe, straight grasper, and straight cutting punch. For the ARTHRO group, training was conducted using a standard 30° surgical arthroscope with light source and camera, LCD monitor, a 5 mm probe, straight grasper, and straight cutting punch.

Performance during both training sessions was evaluated by a second double-blind observer. The following outcome measures were recorded:

1. Time to complete the progressive number-finding task (1–23);
2. Time to complete the labyrinth navigation task (with a maximum of two attempts if the ball fell outside the module);
3. Number of cylinders correctly inserted within 5 min;

The simulated meniscectomy task was included as a practical training exercise to allow participants to practice instrument manipulation and spatial orientation within the simulator. This task was not used as a quantitative outcome measure and, therefore, was not included in the statistical analysis.

Performance improvements between the two training sessions were calculated independently for the ZERO and ARTHRO groups and compared between groups.

### 2.6. Final Evaluation: Shoulder Challenge (SHO-CHA)

**Figure 4:**
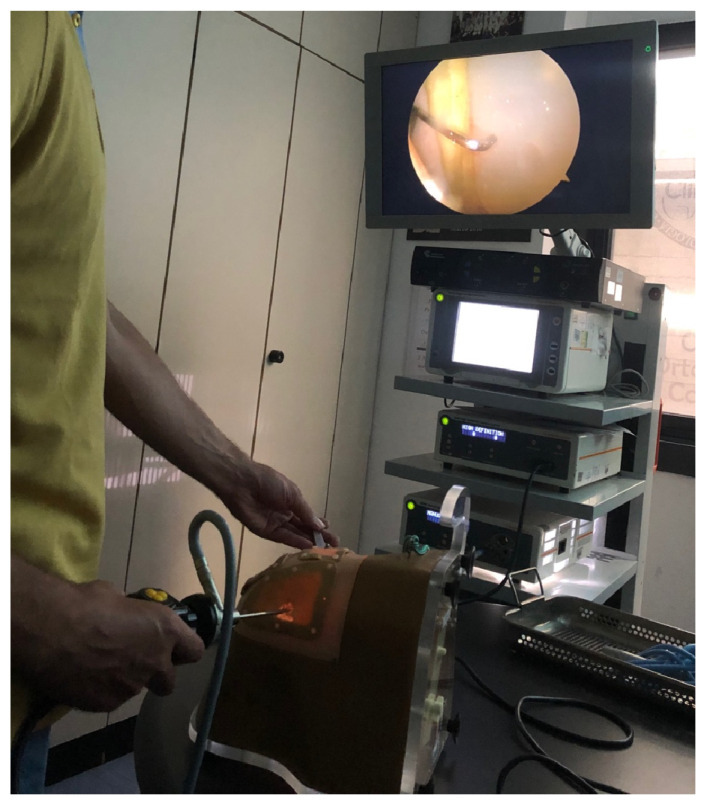
SHO-CHA (Shoulder Challenge) assessment performed using a real arthroscope on an anatomical shoulder model. The image illustrates a participant probing the glenoid labrum during the task execution.

In a final plenary evaluation session, termed the Shoulder Challenge (SHO-CHA), participants from all three groups were assessed by an independent, double-blind orthopedic surgeon with more than five years of experience in shoulder arthroscopy. Assessment was performed using a real surgical arthroscope on a commercially available anatomical shoulder model (ALEX Shoulder Arthroscopy Model; SAWBONES^®^, Linvatec Corporation, Largo, FL, USA) (Figure 4).

During this session, participants were required to identify 14 diagnostic arthroscopic landmarks described by Snyder [42] and perform four additional probing tasks using a standard 30° arthroscope. The diagnostic sequence included the following tasks:

- Tasks 1–14: systematic visualization of glenohumeral and subacromial structures;
- Tasks 15–18: probing maneuvers assessing stability and surface evaluation of key anatomical structures.

For each participant, the total time required to complete the diagnostic sequence and the number of looks away from the monitor (“looks down”) were recorded. A “look down” was defined as any instance in which the participant visually diverted attention from the monitor to the operative field or instruments during task execution. The metric was recorded in real time by the blinded evaluator. The “looks down” metric represents a simplified observational parameter and should be interpreted as a complementary indicator rather than a direct measure of surgical skill. Group comparisons were performed among the ZERO, ARTHRO, and CONTROL groups.

As each participant contributed a single observation for the final task completion time, the dataset consisted of independent observations. Correlation regression analysis was conducted to evaluate potential associations between SHO-CHA completion time and participant-related factors, including the number of arthroscopic procedures observed, the type of manual activity, and the level of interest in arthroscopic surgery.

### 2.7. Statistical Analysis

Continuous variables are presented as mean, standard deviation, minimum, and maximum values. Parametric variables were compared using Student’s *t*-test. The chi-square test was applied to assess group homogeneity in categorical variables. Group comparisons were performed using one-way ANOVA followed by Tukey post hoc tests. The assumptions of normality and homogeneity of variance were assessed using the Shapiro–Wilk test and Levene’s test, respectively. No violations of these assumptions were detected. Effect sizes were calculated using Cohen’s d for pairwise comparisons. Exact *p*-values and 95% confidence intervals (CI) were reported for all primary outcomes. Correlations were assessed using Pearson’s correlation coefficient (ρ) with 95% CI for the correlation coefficient. An a priori sample size estimation was performed for the primary comparative analysis assuming a moderate effect size (Cohen’s d = 0.5), a significance level of 0.05, and a statistical power of 80%. A moderate effect size was selected based on conventional recommendations for exploratory educational intervention studies in the absence of robust preliminary data. Under these assumptions, the estimated minimum sample size was approximately 30 participants, which was met by the final cohort of 33 participants included in the study.

The threshold for statistical significance was set at *p* < 0.05. Statistical analyses and graphical representations (box plots and histograms) were performed using Microsoft Excel (version 16.85) and SPSS software (IBM, version 29).

## 3. Results

### 3.1. Descriptive Analysis

A total of 33 orthopedic residents were included in the study. Overall, 72.7% of participants were male, with a male-to-female ratio of 24:9. The mean age was 28.9 ± 2.6 years (range 25–36). Nineteen residents were postgraduate year I (PGY-I, 57.6%), ten were PGY-II (30.3%), and four were PGY-III (12.1%).

Regarding previous exposure to arthroscopy, 57.6% of participants reported having never observed an arthroscopic procedure, 12.1% reported having observed between 5 and 10 procedures, and 30.3% reported having observed more than 10 procedures. Concerning manual activities, 30.3% reported no regular manual activity, 51.5% reported sports involving hand use or traditional manual activities, and 18.2% reported engagement in digital arts or manual hobbies.

Participants were randomly assigned to the three study groups. The ZERO group included 12 participants (mean age 29.3 ± 3.6 years; range 26–36; M:F = 11:1), the ARTHRO group included 11 participants (mean age 28.4 ± 1.7 years; range 25–31; M:F = 10:1), and the CONTROL group included 10 participants (mean age 29.1 ± 2.1 years; range 27–33; M:F = 3:7). No statistically significant differences were observed among groups for age, postgraduate year, number of arthroscopic procedures observed, or type of manual activity, except for the male-to-female ratio in the CONTROL group (*p* < 0.01) (Table 1).

### 3.2. Training Task Results

Performance outcomes from the two training sessions (Test 1 and Test 2) conducted on the FAST-like workstation are summarized in Table 2. Both the ZERO and ARTHRO groups demonstrated an overall statistically significant improvement between test and retest for all evaluated tasks (paired test–retest analysis, *p* < 0.05), indicating effective skill acquisition through hands-on training.

The cylinder insertion task did not demonstrate statistically significant differences between test and retest when analyzed independently within each group. When comparing the overall task completion times between groups, the ARTHRO group showed faster performance than the ZERO group only in the number-finding task (*p* = 0.033). No other significant differences were observed between the two hands-on training modalities. Effect sizes for task improvement showed moderate to large training effects. In the ZERO group, Cohen’s d values were 1.23 for the numbers task, 0.83 for the labyrinth navigation task, and 0.74 for the cylinder insertion task. In the ARTHRO group, effect sizes were 0.66, 1.56, and 0.68, respectively, indicating moderate to very large improvements between the two training sessions. Between-group comparisons showed moderate effect sizes (Cohen’s d ranging from 0.53 to 0.63).

### 3.3. Shoulder Challenge (SHO-CHA) Results

**Figure 5:**
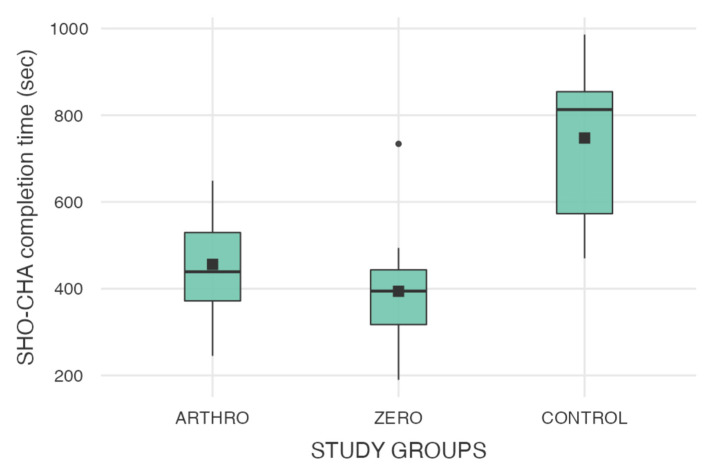
Distribution of SHO-CHA task completion times across the three study groups (ARTHRO, ZERO, CONTROL). The boxplots display the median, interquartile range, and range of values, with individual outliers represented as separate points. Completion time is reported in seconds.

**Figure 6:**
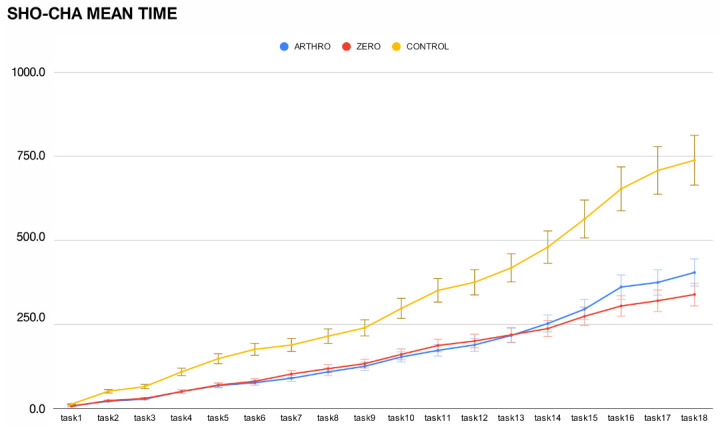
Linear graph showing mean time (s) progression through the different tasks of SHO-CHA (shoulder challenge), comparing three groups included in the study (Yellow line: CONTROL-group, red line: ZERO group, Bue line: ARTHRO group).

Results from the final Shoulder Challenge (SHO-CHA) assessment are reported in Table 3. The mean SHO-CHA completion time was 394.1 ± 140.7 s for the ZERO group (n = 12), 456.1 ± 123.2 s for the ARTHRO group (n = 11), and 745.5 ± 190.7 s for the CONTROL group (n = 10) (Figure 5 and Figure 6). The mean number of looks down was 11.1 ± 2.9 for the ZERO group, 12.6 ± 2.3 for the ARTHRO group, and 16.1 ± 2.3 for the CONTROL group.

A statistically significant difference was observed among the three groups for overall SHO-CHA completion time (One-way ANOVA, F (2,30) = 15.6, *p* < 0.001, η^2^ = 0.51). Post hoc analysis demonstrated a significantly shorter completion time for the ZERO group compared with the CONTROL group (mean difference = −351.4 s, 95% CI: −503.4 to −199.4, Cohen’s d = −2.13, *p* < 0.001). The ARTHRO group also outperformed the CONTROL group (mean difference = −289.4 s, 95% CI: −437.4 to −141.4, Cohen’s d = −1.82, *p* < 0.001). No statistically significant differences were found between the ZERO and ARTHRO groups for SHO-CHA completion time (mean difference = −62.0 s, 95% CI: −177 to 53, Cohen’s d = −0.47, *p* = 0.276). No significant differences were observed among groups regarding the number of looks down (*p* = 0.868).

No significant linear correlations were found between SHO-CHA completion time and the number of arthroscopic procedures previously observed (*p* = 0.150) or the level of manual activity reported by participants (*p* = 0.756).

### 3.4. Likert Questionnaire Results

Overall, participant responses indicated a high level of acceptance and perceived usefulness of the Arthrozero simulator. Eighty percent of participants responded “definitely yes” and 20% responded “yes” regarding the usefulness of the simulator for understanding basic arthroscopic principles, accelerating manual skill acquisition, improving confidence with real arthroscopic instrumentation, and enhancing the overall training experience. Participants also expressed strong agreement on the potential integration of the simulator into postgraduate orthopedic training programs.

Regarding the replacement of traditional arthroscopic training before real surgery, 50% of participants responded negatively, 41.7% responded positively, and the remaining participants reported a neutral opinion. All participants in the ZERO group reported that training with the Arthrozero simulator facilitated the final SHO-CHA session using a real surgical arthroscope, particularly with respect to technical handling and tactile perception.

## 4. Discussion

### 4.1. Main Findings

The findings of this study suggest that the home-based Arthrozero simulator may support the acquisition of basic arthroscopic skills and represents a potentially accessible tool for early training. Training with the proposed simulator was associated with significantly better performance compared with the non-trained control group. When compared with training using a real surgical arthroscope, no statistically significant differences in performance were observed.

The effectiveness of arthroscopic simulation systems has been widely reported in the literature, particularly for virtual and augmented reality-based platforms, which have demonstrated strong construct validity and effective transfer of acquired skills to cadaveric and simulated anatomical models [23,24,27,28,30]. However, the translation of simulation-based training into improved intraoperative performance remains a topic of debate, as highlighted by Declerc et al., who reported that direct clinical transfer has not yet been conclusively demonstrated [43].

One of the main barriers to the widespread adoption of high-fidelity arthroscopic simulators is their high acquisition and maintenance costs. These systems are often inaccessible to individual trainees and to residency programs with limited educational budgets. Consequently, traditional arthroscopic training remains largely dependent on mentorship models and participation in costly cadaveric laboratory courses.

This economic gap has stimulated the development of several innovative low-cost arthroscopic training solutions described in the literature, including open-source 3D-printed simulators [44], validated PVC-based shoulder [35] and knee [36] models, low-cost arthroscopic boxes [33], and basic arthroscopic training stations [34,38,39]. Previous cost-comparison analyses have shown that in-house developed arthroscopy simulators may require as little as 8.3% of the recurring costs of commercially available systems, providing a more accessible alternative for simulation-based training, even in settings with limited financial resources or patient-based constraints [45,46].

Consistent with previous reports, the present study confirms that dry laboratory training alone is sufficient to improve fundamental arthroscopic skills such as triangulation, camera orientation, telescoping, periscoping, and instrument manipulation, independently of direct exposure to the operating room environment or high-end simulators. The use of an adaptation of a validated FAST workstation further supports these findings, as this system has demonstrated adequate construct validity for discriminating different levels of arthroscopic skill and for tracking skill progression over time [40,47,48,49], with no significant deterioration in task performance even after prolonged periods of inactivity [50].

While the effectiveness of low-cost arthroscopic stations has been previously demonstrated, a major limitation of many existing systems lies in the type of arthroscope used during training. The Arthrozero simulator was specifically designed to replicate the handling characteristics, camera orientation, grip ergonomics, and tactile feedback of a real surgical arthroscope. In addition to its affordability, with an estimated production cost of approximately 25 EUR per unit, the Arthrozero simulator maintains a high degree of structural and functional similarity to a real surgical arthroscope. Therefore, the proposed system represents not only a low-cost solution but also a technically reliable and scalable training alternative. The present study did not aim to formally validate the optical fidelity of the simulator. Future studies should investigate aspects such as face and construct validity in order to further evaluate the educational effectiveness of the device. In the present study, its potential utility as a low-cost training tool for basic arthroscopic skill acquisition was supported by performance improvements comparable to those obtained with a real arthroscope. The ZERO group demonstrated similar learning curves and final performance outcomes to the ARTHRO group across all training tasks. Improvements observed during the training sessions may partly reflect learning curve effects related to repeated task exposure rather than the training modality alone. In addition, a potential motivational or expectation effect cannot be excluded, as participants aware of participating in simulator training may have been more engaged in the learning process.

Furthermore, results from the final Shoulder Challenge (SHO-CHA) task demonstrated the transferability of skills acquired using the Arthrozero simulator to a more complex anatomical model. Both trained groups significantly outperformed the control group, confirming the applicability of workstation-based training. The SHO-CHA task represents a standardized basic arthroscopic skill assessment rather than a direct replication of a clinical surgical procedure. Its purpose is to evaluate fundamental visuospatial orientation and instrument handling abilities, which are core components of early arthroscopic skill acquisition.

Unlike commonly used arthroscopic assessment tools such as the ASSET score [19] and OSATS, which are examiner-dependent and often rely on video-based analysis [20], the present study focused exclusively on objective performance metrics, including task completion time and number of looks down, to minimize evaluator bias and enhance reproducibility.

Finally, participant feedback collected through the Likert questionnaire demonstrated a high level of acceptance and perceived educational value of the Arthrozero simulator. Participants unanimously agreed on its usefulness for acquiring basic arthroscopic skills and facilitating the transition to real arthroscopic instrumentation, while recognizing that it does not replace traditional operative training. These findings highlight the potential role of Arthrozero as a complementary educational tool within structured arthroscopic training curricula.

Overall, the key strengths of the Arthrozero system include its suitability for home-based training, high similarity to real arthroscopic instrumentation, cost-effectiveness, scalability of training performance, and the possibility of monitoring skill progression in institutions lacking access to commercially available simulators.

### 4.2. Limitations

Several limitations should be acknowledged. First, the relatively small sample size may limit the generalizability of the findings. Although an a priori power analysis was performed to estimate the minimum required sample size for the primary comparison, the overall cohort remains relatively limited and the results should therefore be interpreted with caution. Additionally, the study population reflects the training environment of orthopedic residents in Italy; therefore, larger multicenter and multinational studies are required to confirm the reproducibility of these results across different educational systems.

Second, the final anatomical assessment focused exclusively on the shoulder joint, which allowed a comprehensive 360° arthroscopic evaluation of basic skills. However, assessment of other joints, such as the knee or ankle, may provide additional insights and potentially different performance outcomes. An additional limitation of the present study is the uneven sex distribution among the groups, particularly in the control cohort. Although sex was not considered a primary determinant of technical skill acquisition in this exploratory design, future studies with larger and more balanced samples should account for potential demographic confounders such as sex, training level, prior arthroscopic exposure, and variability in clinical training environments, which may introduce ecological bias. Another limitation relates to the unequal practical training exposure between the intervention groups and the control group, which received only theoretical instruction. In addition, participants were not blinded to their group allocation, which may have introduced motivational or expectation bias during the training process. Additionally, the relatively short training exposure (two sessions) may limit conclusions regarding long-term skill acquisition.

Finally, this study evaluated the effectiveness of Arthrozero exclusively for basic arthroscopic skills and it also did not evaluate long-term skill retention. In addition, external validation of the Arthrozero simulator against established arthroscopic training platforms was not performed and should be addressed in future comparative studies. Further investigations involving more advanced arthroscopic procedures, such as knot tying, meniscal repair, or complex instrument handling on advanced models, are required to assess the simulator’s utility at higher levels of training.

## 5. Conclusions

Arthroscopic surgery requires complex visuospatial and psychomotor skills, including telescoping, periscoping, probing, and instrument manipulation, which are primarily developed through repetitive practice. The findings of this study suggest that an affordable home-based arthroscopic simulator may support the acquisition of basic arthroscopic skills in surgeons in training. The Arthrozero simulator may represent a useful educational tool for structured practice and for monitoring skill progression, particularly in institutions with limited access to conventional arthroscopic training resources.

## Figures and Tables

**Table 1:** Descriptive characteristics of the participants included in the study.

Group/Variable		Total	ZERO Group	ARTHRO Group	CONTROL Group	*p*
Participants (n)		33	12	11	10	/
AGE (mean; min–max/std)		28.9; (25–26)	29.3 (±3.6)	28.4; (±1.7)	29.1 (±2.13)	0.614
M:F		24:9	11:1	10:1	3:7	<0.01
PGY-I (n)		19	8	7	4	0.532
PGY-II (n)		10	2	3	5	
PGY-III (n)		4	2	1	1	
Arthroscopic Procedures watched as an observer: (n, %) (A: less than 1; B: 5–10; C: more than 10)	A	19 (57.6%)	8 (66.7%)	7 (63.6%)	4 (40%)	0.812
	B	6 (18.2%)	1 (8.3%)	2 (18.2%)	3 (30%)	
	C	8 (24.2%)	3 (35%)	2 (18.2%)	3 (30%)	
Kind of Manual activity: (n, %) (A: none; B: Manual sports C: arts, digital, hobbies)	A	10 (30.3%)	4 (33.3%)	5 (45.5%)	1 (10%)	0.107
	B	6 (18.2%)	1 (8.3%)	3 (27.3%)	2 (20%)	
	C	17 (51.5%)	7 (58.3%)	3 (27.3%)	7 (70%)	

**Table 2:** Analysis tab showing mean time with std (standard deviation) among tasks, % (percentage of improvement), and mean differences (diff) between test and re-test of participants for each group. On the bottom difference in task mean time between ZERO and ARTHO-groups.

Group/Training Task		Numbers Finding (Mean Time, Std)	Labyrinth Navigation (Mean Time, Std)	Cylinders Insertion (Numbers of Cylinder, Std)
ZERO-Group	Test1	560.12 (±163.6)	224.10 (±47.9)	4.5 (±1.3)
	Test2	371.83 (±139.8)	188.25 (±38.8)	5.58 (±1.62)
Diff Mean (%); [95% CI]; cohen’s d;		188.33 (18.7%) [59.4–317.2] 1.23	35.8 (47.4%) [−1.87–73.5] 0.83	−1.08 (24%) [−2.3–0.2] 0.74
*p*		0.006	0.04	0.136
ARTHRO Group	Test1	368.9 (±126.2)	245.10 (±101.3)	5.33 (±2.6)
	Test2	290.67 (±108.5)	118 (±59.8)	6.75 (±1.4)
Diff Mean; (%); [95% CI]; cohen’s d;		78.25 (33%) [−21.4–177.9] 0.66	127.10 (7.7%) [54.6–199.6] 1.56	−1.42 (35.8%) [−3.1–0.36] 0.68
*p*		0.118	0.002	0.112
Zero-Group Overall		466 (±177.2)	195.65 (±61.3)	5.04 (±1.5)
Arthro-Group Overall		368.92 (±123.5)	245.1 (±98.6)	6.04 (±2.1)
Diff Mean; [95% CI]; cohen’s d;		97.8 [185.8–8] 0.63	49.5 [−0.42–99.3] 0.61	1.00 [−0.1–2.10] 0.53
*p*		0.033	0.176	0.07

**Table 3:** Results of the SHO-CHA (Shoulder Challenge) final assessment across study groups. Groups are presented in rows. Columns report the mean SHO-CHA completion time (seconds), the mean differences in completion time compared with the ZERO group, the number of “look-downs” (instances in which participants diverted their gaze from the monitor), and the corresponding differences relative to the ZERO group.

Group	N	SHO-CHA Time (Mean; Std)	SHO-CHA Time (Min–Max)	Δ SHO-CHA Mean Time vs. ZERO-Group (Mean Diff (s); [95% CI] (s); Cohen’s d)	*p*	Look Down (Mean, Std)	Δ Look Down Mean Number, vs. ZERO-Group (Mean Diff; [95% CI]; Cohen’s d)	*p*
ZERO	12	394.1 (±141)	190–734	/	/	11.1 (±2.9)	/	/
ARTHRO	11	456.1 (±123)	245–649	−62; [−177, 53]; −0.47	0.276	12.6 (±2.3)	1.55 [−0.76, 3.87]; 0.58	0.178
CONTROL	10	745.5 (±192)	470–986	−351.4; [−503.4, −199.4]; −2.13	<0.001	16.1 (±2.3)	5.02 [2.64, 7.40]; 1.88	<0.001
overall *p*	/	<0.001	/	/		0.868		

## Data Availability

The datasets presented in this article are not readily available, the data are part of an ongoing study. Requests to access the datasets should be directed to first author M.M.

## Abbreviations

The following abbreviations are used in this manuscript:

ANOVA	Analysis of Variance
ASSET	Arthroscopic Surgical Skill Evaluation Tool
FAST	Fundamentals of Arthroscopic Surgery Training (workstation)
PGY	Post-Graduate Year
PLA	Polylactic acid
SD	Standard Deviation
SHO-CHA	Shoulder Challenge (final assessment task in the study)
SPSS	Statistical Package for the Social Sciences
USB	Universal Serial Bus
